# Depressive Symptom Network Associated With Comorbid Anxiety in Late-Life Depression

**DOI:** 10.3389/fpsyt.2019.00856

**Published:** 2019-11-20

**Authors:** Min Ho An, Soon Sang Park, Seng Chan You, Rae Woong Park, Bumhee Park, Hyung Kyoo Woo, Han Ki Kim, Sang Joon Son

**Affiliations:** ^1^Ajou University School of Medicine, Suwon, South Korea; ^2^Department of Biomedical Informatics, Ajou University School of Medicine, Suwon, South Korea; ^3^Department of Psychiatry, Ajou University School of Medicine, Suwon, South Korea

**Keywords:** depression, geriatric (aging), anxiety, network analysis, psychopathology

## Abstract

**Background:** Depression and anxiety are the most common comorbid psychiatric disorders in the elderly. Psychiatrists have been reporting worsened depression symptoms and prognosis by comorbid anxiety symptoms. However, it is still unclear how anxiety affects the course of depression in the elderly. The aims of this study are (1) to identify the symptom network in late-life depression (LLD), and (2) to examine the role of anxiety in LLD with a network perspective.

**Methods:** The study analyzed 776 community-based participants who were clinically diagnosed with depression and enrolled in Suwon Geriatric Mental Health Center. Network analysis was used to investigate the relationships between the symptoms of the Montgomery–Åsberg Depression Rating Scale (MADRS). The depression sample was divided into groups of low and high anxiety according to the Beck Anxiety Index. Propensity score matching (PSM) was used to minimize the effects of depression severity on the network. Network comparison test (NCT) were carried out to compare the global connectivity, global strength, and specific edge strength between the two subgroups.

**Results:** Reported sadness, pessimistic thinking, and suicidal ideation are the core symptoms of LLD in terms of node strength. The MADRS sum score [mean (SD) 28.10 (9.19) vs 20.08 (7.11); *P* < .01] was much higher in the high anxiety group. The NCT before PSM showed the high anxiety group had significantly higher global strength (*P* < .01). However, the NCT after PSM did not reveal any statistical significance both in global structure (*P* = .46) and global strength (*P* = .26). A comparison between centrality indices showed a higher node strength of vegetative symptoms in the high anxiety group and this also remained after PSM.

**Conclusion:** Based on the statistical analysis, anxiety worsens the severity of depression in the elderly. However, NCT after PSM revealed comorbid anxiety does not change the global structure and strength of the depression symptom network. Therefore, anxiety may affect LLD in a way of worsening the severity, rather than changing psychopathology. Additionally, the study revealed the centrality of vegetative symptoms was low in LLD but increased substantially in patients with comorbid anxiety.

## Introduction

### Depression in Late-Life

Late-life depression (LLD) poses a global burden (1). Approximately 10% of older population visiting primary care facilities manifest clinically significant depression (2). Patients with LLD exhibit chronic medical disorders or persistent insomnia, functional decline, or social isolation (3). Untreated LLD is associated with a poor quality of life, challenges with social and physical functioning, poor adherence to treatment, and worsening of chronic medical ailments (4). Although patients with LLD have more comorbidities and are more disabled than younger patients with depression (5, 6), LLD is often unrecognized by general practitioners due to the atypical and vague presentations of LLD, which can result in under-treatment of the disease (7). Therefore, early identification of symptom presentations and understanding symptom relations of LLD may lead to a better prognosis, However, the manifestation of depression symptoms in older adults, especially the relationship between different symptoms has yet to be investigated. In this study, our goal was to find the pattern of symptom presentation and relationship between symptoms of depression in the elderly using network analysis.

### Depression Comorbid With Anxiety in the Elderly

Depression and anxiety are two of the most common psychiatric issues in the elderly population (8, 9) Recognition and management of depression and anxiety in elderly is crucial given the health care burden, increased risk for medical illness and disability, and higher mortality rates (10, 11). It is generally known that anxiety and depression share a common diathesis. For instance, both are associated with a negative affect, stressful life events, and impaired cognitive processes suggesting a common biological and genetic predisposition (12). However, anxiety and depression are not similar states or conditions. Studies have reported differences in their heterogeneous traits, adaptive functions and associations with regulatory processes, positive affect, and motivation or complex cognitive processes (12). The term “anxious depression” refers to major depressive disorder with clinically significant but subsyndromal anxiety symptoms (13). Compared with patients diagnosed with depression alone, patients with anxiety show a higher rate of suicidal ideation and history of suicide attempts (14–17). A previous systematic reviews and meta-analyses showed that the rate of suicide was higher in patients with any type of anxiety disorder except obsessive compulsive disorder (18–20). From this background (14–20), we hypothesized that comorbid anxiety may alter the psychopathology of depression in the elderly which may lead to a worse prognosis.

### Network Analysis

In the last several years, network analysis has received more attention in psychiatric research (21). In this field, psychological behavior is explained by complex interaction of psychological and environmental factors (22). The network analysis suggests a symptom can be the cause of other symptoms, leading to consistent profiles or syndromes (21, 23). The networks consist of nodes and edges representing observed variables (often symptoms of disease) and statistical relevance, respectively (22). A typical method of assessing the importance of nodes in the network is computing centrality indices (24, 25). There are three components in centrality indices: node strength (quantification of direct connectivity between each nodes), closeness, (quantification of indirect connectivity between each nodes), and betweenness (quantification of importance of a node in the average path between two other nodes) (22, 24, 25). In this sense, network analysis is an excellent methodology to provide intuitive explanation of psychopathology (26, 27). To our knowledge, this is the first study investigating the role of anxiety in depression among the elderly in network perspective.

## Methods

### Study Sample

All the study subjects were selected from an ongoing longitudinal survey investigating the overall mental status of the elderly aged over 60 in Suwon City, South Korea (28). All participants, clinically diagnosed with depression and voluntarily visited Suwon Geriatric Mental Health Center (SGMHC) in Korea, were recruited from community. The survey was designed by psychiatrists of Ajou University Hospital Department of Psychiatry. The survey contained personal information including age, sex, marital status, number of years of education, family composition, history of illness, and so forth. Additionally, the survey included various scales associated with mental health such as Korean version of Montgomery–Åsberg Depression Rating Scale (MADRS), Beck Anxiety Index (BAI), and others. In this study, we used the cross-sectional data of 1,137 participants during their first visits from 2016 to 2019. The survey was conducted following written informed consent.

### Selection of Participants for Baseline Network

The MADRS is a useful psychiatric assessment tool often used in clinical studies and trials for selection of subjects with a depressive mood (29). MADRS comprises 10 items and each item is scored ranging from 0 (not at all) to 6 (very severe) according to the participant’s symptom severity (30). Additionally, MADRS is a valid tool for the determination of ongoing state of depression and a score of 10 is an appropriate threshold for the selection of subjects with remission (31). Therefore, 848 depressed participants were selected with an MADRS score of 10 or higher to include participants with prominent depressive symptoms without remission at the first visit. During the recruitment process, the participants who showed substantial cognitive impairment and had definite dementia were excluded based on the clinical decision made by psychiatrists of Ajou University Hospital. Participants with a history of bipolar disorder [5], schizophrenia [8], and other mental disorders [59] were excluded. Subjects with past history of anxiety disorder were not excluded, because diagnostic categories of anxiety disorder overlap with that of mood disorders (32). Finally, 776 participants with marked depression symptoms were selected. The overall flowchart outlining the study enrollment is shown in **Supplementary Figure 1**.

### Selection of Low and High Anxiety Cases of Depression

BAI is a useful tool to reliably measure the extent of anxiety, and is especially designed for use in psychiatric populations (33). The BAI consists of 21 items rated according to a 4-point Likert scale from 0 (not at all) to 3 (it bothered me a lot) (33). The role of BAI as a diagnostic tool for anxiety disorder is still disputed (34). However, the BAI is a valid test for the measurement of overall anxiety level in both non-clinical and clinical participants (35). Therefore, in this study, the Korean version of the BAI was used as a questionnaire to determine the overall degree of individual anxiety in subjects who already had depression. The recommended clinical interpretation of BAI is as follows: 0–7 suggests minimal anxiety, 8–15 suggests mild anxiety, 16–25 suggests moderate anxiety, and 26–63 suggests severe anxiety (36, 37). A previous study demonstrated that participants who scored 16 or higher on BAI were categorized as high anxiety patients who needed clinical intervention (36). In this study, the BAI score of 16 was set as a cutoff value to divide 776 participants into two groups: low anxiety (minimal to low anxiety) and high anxiety (moderate to severe anxiety) (*N* = 462 vs *N* = 314).

### Control of Depression Severity Using Propensity Score Matching

Even if the networks of high and low anxiety group showed the difference, the cause for such difference can still be unclear because comorbid anxiety itself may have affected the severity of depression (38, 39). For this reason, subjects were matched from each high and low anxiety group using propensity score matching (PSM) based on MADRS sum score to remove the effect of depression severity. The low anxiety group was set as a control group, and high anxiety group was set as a treatment group in PSM methodology. Additionally, a few covariates such as years of education, gender, and age were controlled to minimize the confounding bias. Each value of covariates was converted to propensity score by logistic regression and summed to calculate total propensity score (40). “Nearest neighbor matching” method was used, and subjects who have the least difference in total propensity score were paired in 1:1 ratio (40). The caliper was set to.25 to minimize the mean squared error (MSE) (41). Finally, unmatched samples were discarded and same numbers of participants (*N* = 199) were allocated after PSM. This procedure was conducted by R-package *MatchIt* (42).

## Statistical Analysis

### Network Estimation

Gaussian graphical models were developed to obtain the MADRS item scores of 776 participants with depression based on polychronic correlation using R software package, *qgraph* (43). Nodes of network are composed of 10 items of MADRS, and all items were matched with depression symptoms according to DSM-5 (44) (**Supplementary Table 1**). Each network was estimated separately using the MADRS symptom node. The least absolute shrinkage and selection operator (LASSO) procedure was used in each network to visualize only the significant edges in the network (45). Network edge weight accuracy was evaluated by bootstrapping with edge strengths’ 95% CIs using R software package, *bootnet* (22). We computed node strength compared with connection to other nodes to determine the centrality indices with γ = 0. Statistical analyses were performed using R-package version 3.0.2. We present one estimates of centrality to investigate the association of the 10 symptoms of the LLD symptom network: strength (46, 47).

**Table 1:** Baseline characteristics of patients with late-life depression.

Variable	Marked Depression with MADRS > = 10 (N = 776)	
	Mean	SD
Age	73.87	8.06
Education years	6.16	4.52
MADRS sum score	23.32	8.92
Item #1 (Apparent Sadness)	2.64	1.39
Item #2 (Reported Sadness)	3.13	1.43
Item #3 (Inner tension)	2.69	1.34
Item #4 (Reduced sleep)	2.70	1.88
Item #5 (Reduced appetite)	1.95	2.04
Item #6 (Concentration difficulties)	1.76	1.40
Item #7 (Lassitude)	2.01	1.55
Item #8 (Inability to feel)	2.36	1.46
Item #9 (Pessimistic thought)	2.29	1.35
Item #10 (Suicidal thought)	2.08	1.54
BAI sum score	15.20	12.02

MADRS indicates Montgomery–Åsberg Depression Rating Scale; BAI, Beck Anxiety Inventory.

### General Differences

Independent two-sample t-test, paired t-test, and independent two-population proportion tests were performed to compare the general differences between the two groups. The significance level for all analyses was α = .05. Statistical analyses were performed using R-package, version 3.0.2.

### Differences in Overall and Local Connectivity

Overall connectivity was calculated using R-package *Network Comparison Test* (NCT). NCT is a two-tailed permutation test in R-package used to calculate the difference between two networks *via* repeated and random regrouping of individuals (48). NCT is a useful tool for the comparison of two independent, cross-sectional data sets and to investigate the differences in global structure (i.e., which symptoms were most strongly associated with one another), global strength (i.e., difference in sum of edge weights of two networks), and specific edge strength (48). Local connectivity was based on the absolute value of node strength determined using R-package *qgraph*.

## Result

### Depression Symptom Network of Elderly

**Figure 1:**
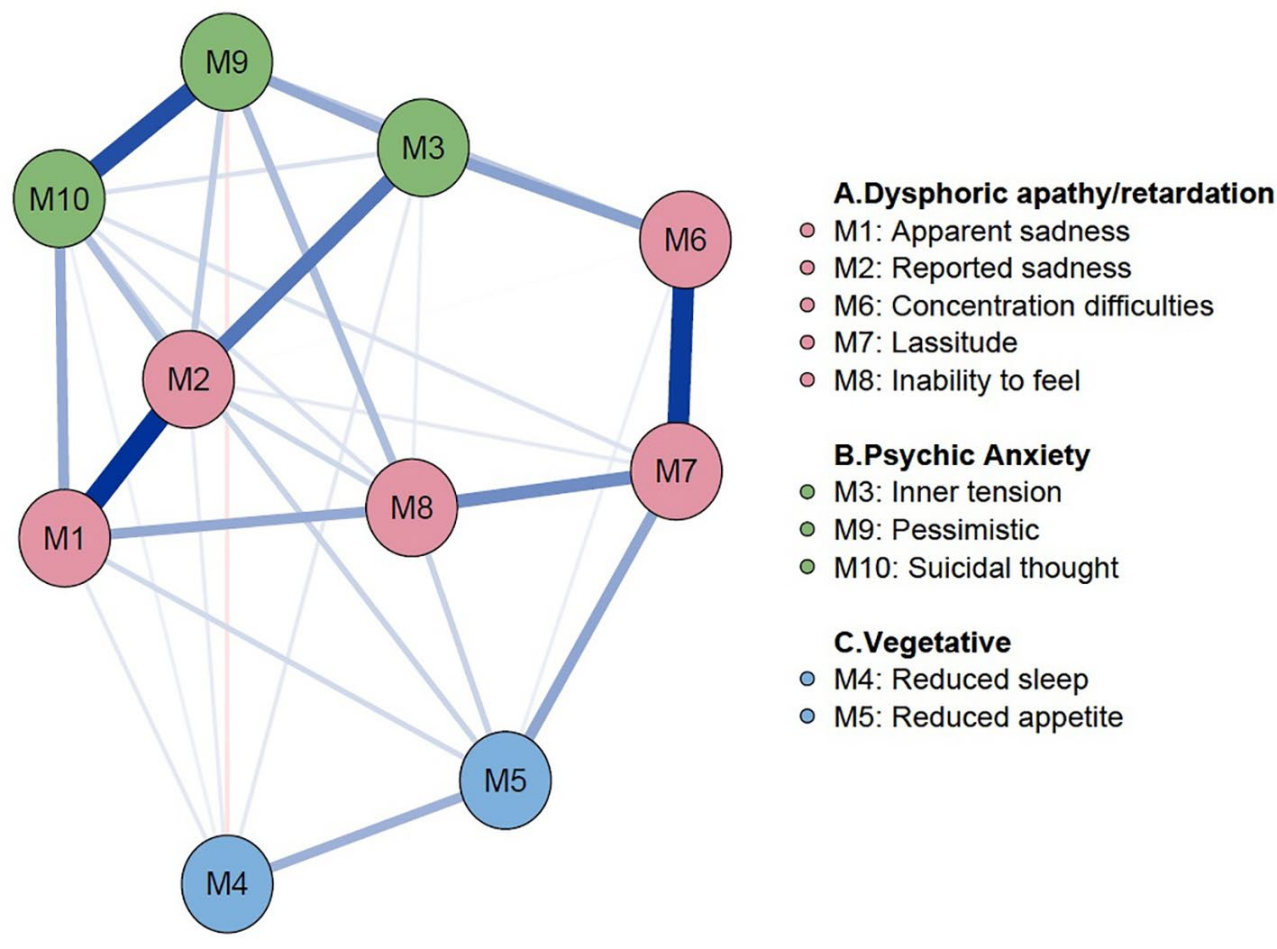
Baseline network of late-life depression. Network Analysis on 776 patients with late-life depression was performed. The symptoms were connected closely to each other within the known subgroups of the MADRS symptoms. The color of each node represents symptoms in MADRS. Color in node represents the subgroup of each symptoms, color of subgroups are as follows: **(A)** (red color): Dysphoric apathy/retardation symptom group; **(B)** (green color): Psychic anxiety symptom group; **(C)** (blue color): Vegetative symptom group. Edges in blue color represent proportional relationships and edges in red color represent inverse relationships. The magnitude of color represents the degree of the relationship between symptoms. MADRS, Montgomery–Åsberg Depression Rating Scale.

**Figure 2:**
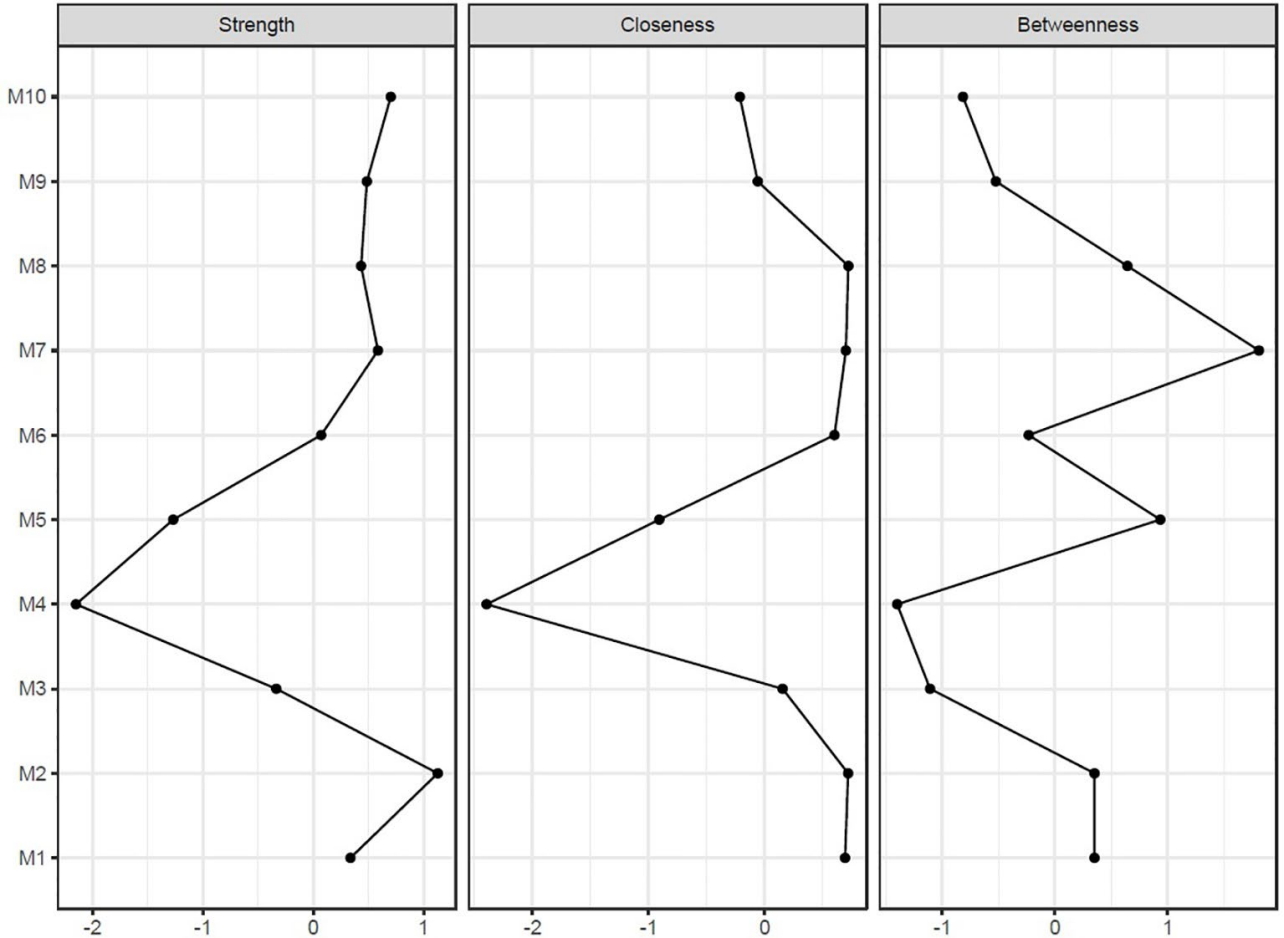
Centrality indices of baseline network. Horizontal axis represents the raw value of centrality indices which is the degree of involvement of the focal node to other nodes in network. Vertical Axis represent each symptoms of depression. Node strength quantifies how well a node is directly connected to other nodes. Closeness quantifies how well a node is indirectly connected each other. Betweenness quantifies how important a node is in the average path between two other nodes.

General demographic information of 776 participants with depression and psychiatric scales including MADRS and BAI are shown in **Table 1**. In the sample, the mean (SD) age was 73.87 (8.06) years, and the mean (SD) MADRS score was 23.32 (8.92). Using this sample, the depression symptom network of the elderly was estimated according to the items listed in MADRS (**Figure 1**). In this network, blue edges represent positive relationships, and a red edge denotes a negative relationship between symptoms. If nodes are strongly connected, the color of the edge is thicker and more vivid. In our results, the edges of *concentration difficulties—lassitude*, *apparent sadness—reported sadness*, and *pessimistic thought—suicidal thought* are strong connections. However, the edges between *reduced sleep—pessimistic thought* showed a negative relationship. *Reported sadness*, *pessimistic*, *suicidal thought,* and *lassitude* were shown as core symptoms in terms of centrality indices; strength, closeness, and betweenness (**Figure 2**). However, in the vegetative symptom group, *reduced sleep* and *reduced appetite* showed the lowest node strength. Bootstrapped 95% CIs of network strength and network stability are presented in **Supplementary Figure 2**.

### Addition of Anxiety Node on the Depression Symptom Network

**Figure 3:**
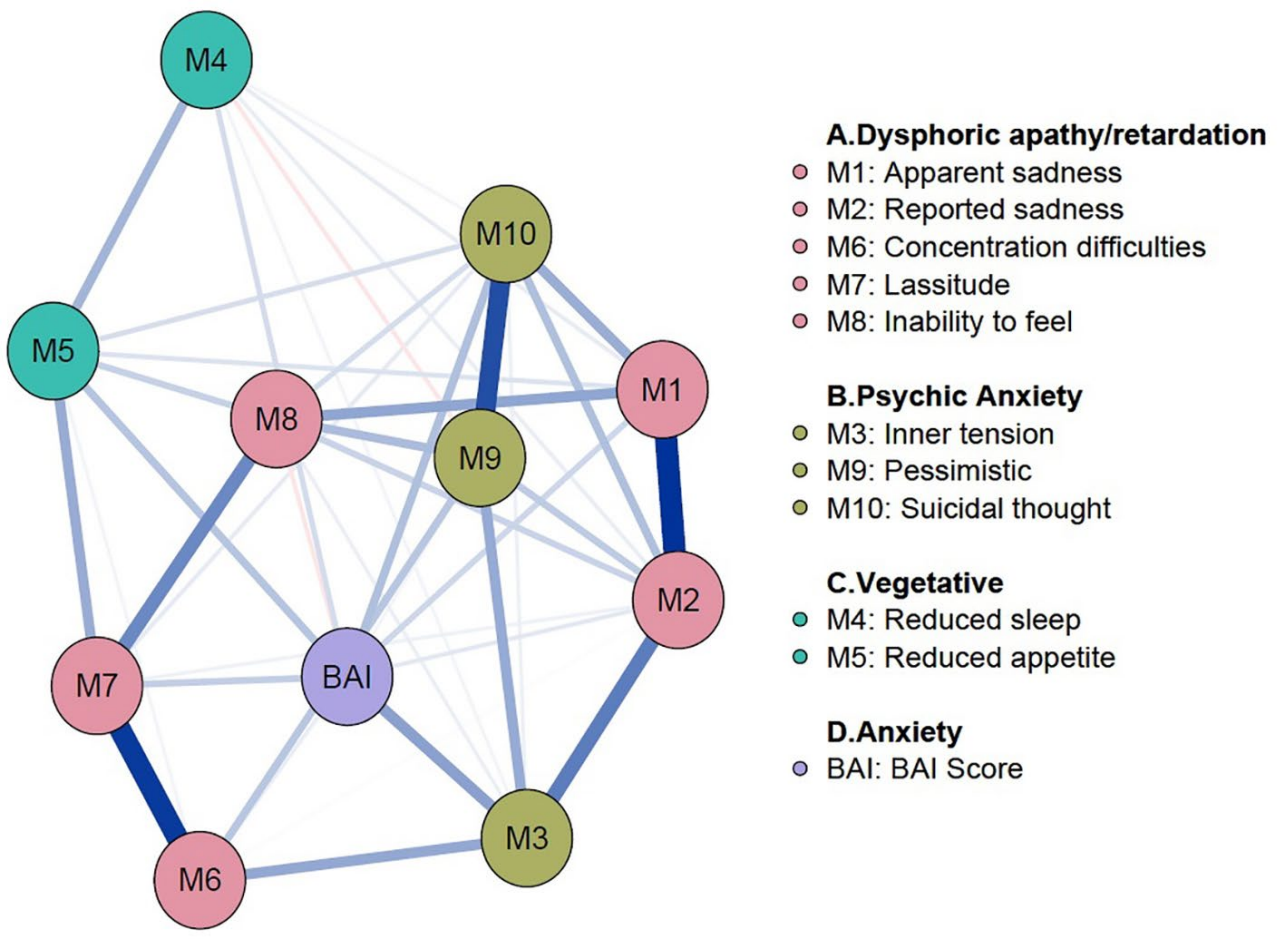
Addition of an anxiety node to the depression symptom network. The anxiety node was added to the baseline network of LDD to see the effects of anxiety on symptoms of depression. The BAI sum score was used to compute and add the anxiety node. The anxiety node is located relatively at the center and has positive connections with all items of MADRS except *inability to feel*. BAI, Beck Anxiety Inventory; LDD, Late-life Depression; MADRS, Montgomery–Åsberg Depression Rating Scale.

To analyze the effects of anxiety on depression symptoms in the elderly, anxiety node was added to the depression symptom network (**Figure 3**). The BAI sum score was used to generate an anxiety node. In the network, an anxiety node is located relatively at the center and showed positive connections with all items of MADRS except *inability to feel*. Among them, edges with *inner tension*, *reduced appetite*, *concentration difficulties*, and *suicidal thought* showed noticeably strong connection with anxiety node. Centrality indices of the network are shown in **Supplementary Figure 3**. Bootstrapped 95% CIs of network strength and network stability are shown in **Supplementary Figure 4**.

### General Differences Before and After Propensity Score Matching

Demographic differences between low and high anxiety before and after PSM are shown in **Table 2**. Before PSM, the t-test results indicated that the age of subjects in the high anxiety group was lower than the low anxiety group [mean (SD) 71.99 (9.63) vs 74.87 (7.39); *P* < .01]. However, the MADRS sum score [mean (SD) 28.10 (9.19) vs 20.08 (7.11); *P* < .01] and BAI sum scores [mean (SD) 27.15 (9.30) vs 7.08 (4.58); *P* < .01] were higher in high anxiety group. Before PSM, 322 out of 462 subjects were female in low anxiety group. On the other hand, 240 out of 314 subjects were in high anxiety group. The gender ratio between two groups showed significant difference (69.70% vs 76.43%; *P = .048*). After PSM, 151 and 150 female subjects were selected out of total 199 subjects in low and high anxiety group respectively (75.88% vs 75.38%; *P = 1.00*). The years of education did not show statistically significant difference [mean (SD) 5.79 (4.52) vs 6.40 (4.51); *P* = .07]. After PSM, the p-values from paired t-test of all items except *inability to feel* and BAI score were greater than.05. Especially, p-value of MADRS sum score [mean (SD) 25.28 (8.14) vs 24.00 (7.60)] was .11, which suggests appropriate matching was done to eliminate the effect of depression severity.

**Table 2:** Demographic features before and after propensity score matching.

Variable	Before Propensity Score Matching				*p*-value	After Propensity Score Matching				*p*-value
	Low Anxiety (BAI < 16, n = 462)		High Anxiety (BAI ≥ 16, n = 314)			Low Anxiety (BAI < 16, n = 199)		High Anxiety (BAI ≥ 16, n = 199)		
	Mean	SD	Mean	SD		Mean	SD	Mean	SD	
Age	74.87	7.39	71.99	9.63	<0.01*	73.34	7.27	73.08	9.07	0.74**
Education years	6.40	4.51	5.79	4.52	0.070*	5.79	4.43	5.69	4.60	0.82**
MADRS sum Score	20.08	7.11	28.10	9.19	<0.01*	24.00	7.60	25.28	8.14	0.11**
Item #1 (Apparent Sadness)	2.29	1.30	3.14	1.37	<0.01*	2.74	1.36	2.80	1.33	0.65**
Item #2 Reported Sadness)	2.76	1.34	3.68	1.38	<0.01*	3.16	1.34	3.31	1.30	0.29**
Item #3 (Inner tension)	2.10	1.19	3.04	1.35	<0.01*	2.46	1.22	2.70	1.24	0.052**
Item #4 (Reduced sleep)	2.44	1.89	3.04	1.80	<0.01*	2.80	1.94	2.86	1.79	0.73**
Item #5 (Reduced appetite)	1.60	1.38	2.50	2.65	<0.01*	1.86	1.50	2.28	3.13	0.089**
Item #6 (Concentration difficulties)	1.49	1.32	2.15	1.42	<0.01*	1.76	1.39	1.95	1.31	0.17**
Item #7 (Lassitude)	1.69	1.46	2.48	1.57	<0.01*	2.24	1.51	2.24	1.44	0.97**
Item #8 (Inability to feel)	2.13	1.39	2.70	1.51	<0.01*	2.66	1.40	2.37	1.38	0.040**
Item #9 (Pessimistic thought)	1.99	1.24	2.73	1.38	<0.01*	2.26	1.27	2.44	1.30	0.15**
Item #10 (Suicidal thought)	1.64	1.43	2.73	1.48	<0.01*	2.07	1.51	2.35	1.41	0.060**
BAI sum score	7.08	4.58	27.15	9.30	<0.01*	8.15	4.73	25.96	9.24	<0.01**

MADRS indicates Montgomery–Åsberg Depression Rating Scale; BAI, Beck Anxiety Inventory. *p-value is obtained by t-test. **p-value is obtained by paired t-test.

### Differences in Global Structure and Strength

**Figure 4:**
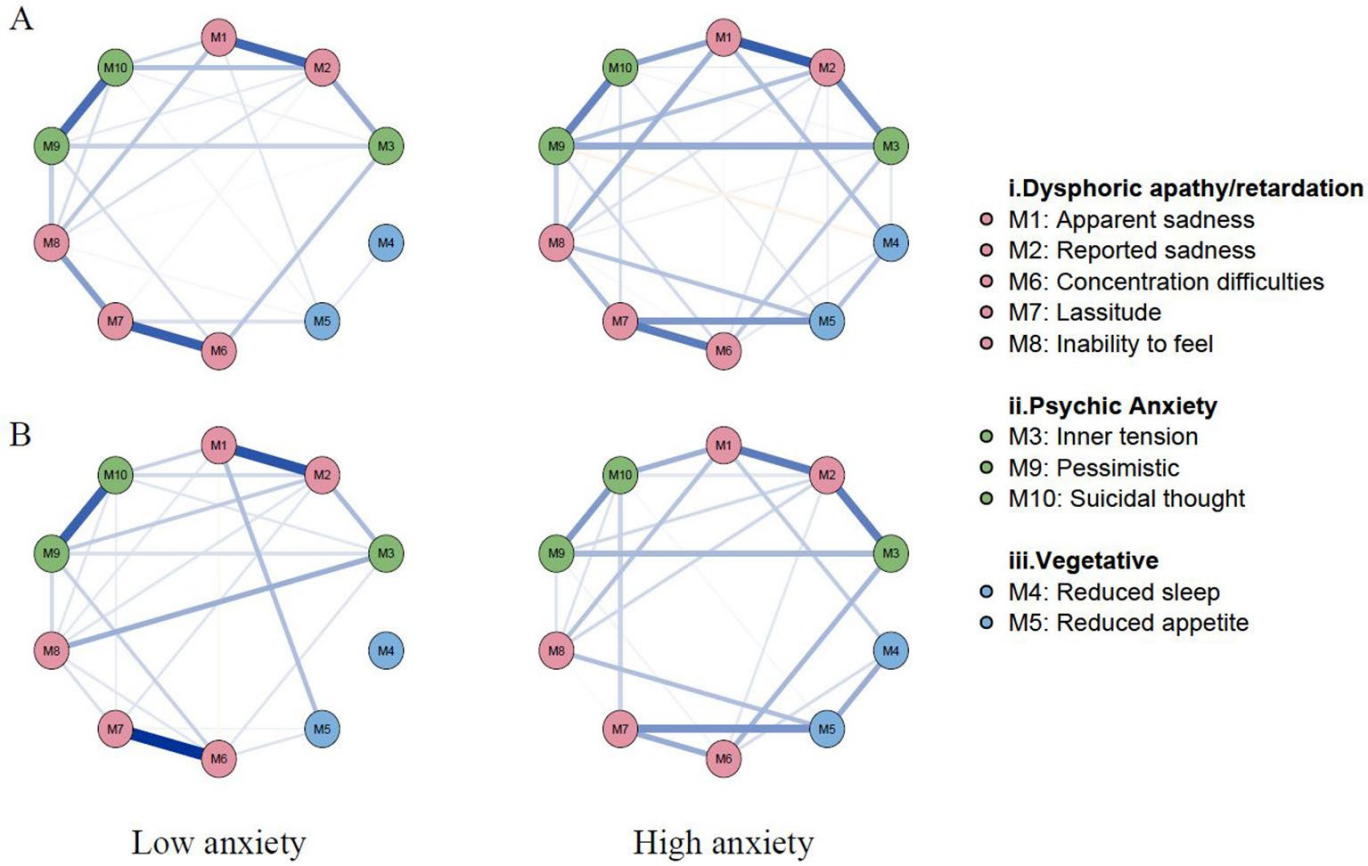
Network structures with low vs high anxiety before and after propensity score matching. **(A)** Network structures before propensity score matching; **(B)** Network structures after propensity score matching. Overall connectivity was increased in the high anxiety group before propensity score matching. Remarkable changes in the high anxiety group are newly formed edges between *lassitude [M7]— suicidal thought [M10]* and *apparent sadness [M1] —reduced sleep [M4]*. Edges in blue color represent proportional relationships and edges in red color represent inverse relationships. The magnitude of color represents the degree of the relationship between symptoms.

**Figure 5:**
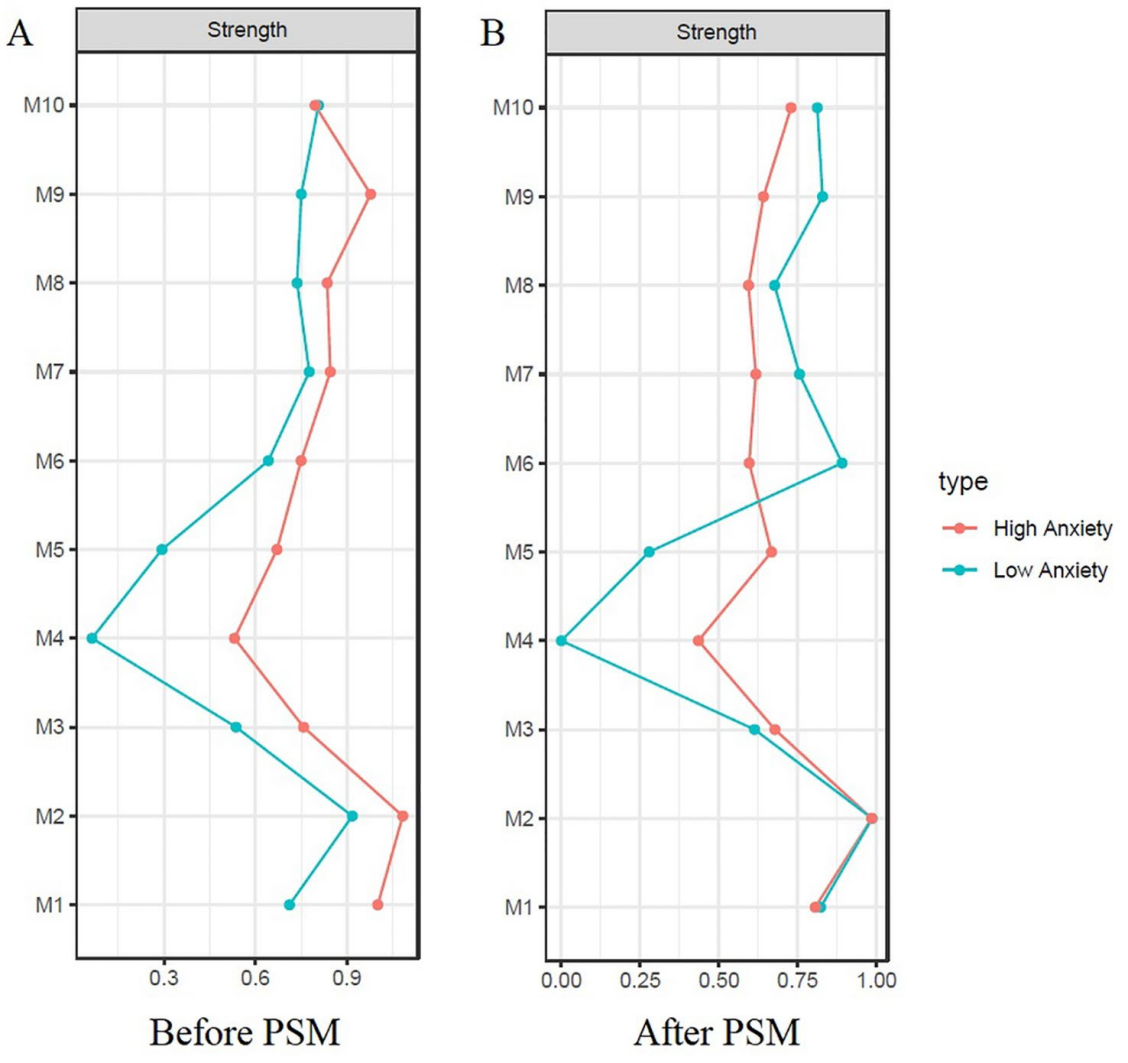
Centrality indices (strength) of low vs high anxiety before and after propensity Score matching. **(A)** Before propensity score matching; **(B)** After propensity score matching. Remarkably higher node strength is observed in all nodes except *suicidal thought [M10] *in the network* before* PSM **(A)**. After PSM, such order was reversed except for the centrality indices of inner tension [M3] and vegetative symptoms (reduced sleep [M4] and reduced appetite [M5]) **(B)**. The horizontal axis represents node strength which is the degree of connection of a node to all other nodes in network. The vertical Axis represent each symptoms of depression.

Comparison of gross network structure between low and high anxiety groups is shown in **Figure 4**. The networks before PSM are indicated in **Figure 4A**. Grossly, most of the edges were strengthened in the high anxiety group. The striking difference involved new edges in the high anxiety group: involving *lassitude—suicidal thought* and *apparent sadness—reduced sleep.* There was also a negative edge in high anxiety group; *reduced appetite—pessimistic.* The networks after PSM are presented in **Figure 4B**. Although the gross pattern is similar to **Figure 4A**, the overall network connection was sparse in the high anxiety group after PSM compared to the high anxiety group before PSM. Comparison of centrality indices showed that the node strength of high anxiety group was significantly higher in all nodes except *suicidal thought* before PSM (**Figure 5A**). After PSM, the higher value of centrality indices including *inner tension and* vegetative symptoms (*reduced sleep and reduced appetite)* was maintained (**Figure 5B**). The NCT before PSM revealed a significant difference in global strength (*P* = .002) but not in global structure (*P* = .46). However, the NCT after PSM revealed no statistical difference between global structure (*P* = .46) and strength (*P* = .26). Bootstrapped 95% CIs of network strength and network stability are shown in **Supplementary Figure 5**.

## Discussion

Utilizing the network approach, the symptom network of depression was analyzed in elderly subjects attending SGMHC, and differences in the network in the presence of comorbid anxiety symptoms. To our knowledge, this study is the first to apply a network approach to depression analysis in elderly subjects, as well as the first to explore the effects of anxiety using this method. In the baseline network of depressive symptoms, the MADRS symptoms clustered together, according to their subgroups. Interestingly, the result was consistent with a previous study of Parker et al. (49); The 10 items of MADRS were regrouped into three analytic models: factor 1*, dysphoric apathy/retardation;* factor 2, *psychic anxiety;* and factor 3, *vegetative symptoms*. Symptoms of each subgroup were located closely to each other and showed stronger connections. The strongest edges existed between *reported sadness—apparent sadness, concentration difficulties—lassitude, pessimistic thinking—suicidal thought*, which represent edges within subgroups. Symptoms within the vegetative subgroup were sparsely connected, showing that this group played a smaller role in elderly depression. Parker et al. (49) stated that dysphoric apathy/retardation and psychic anxiety more likely reflected subtypes of LLD rather than vegetative symptoms (49).

By adding an anxiety node to the baseline depression network, anxiety showed interactions with various symptoms of depression in addition to symptoms of “psychic anxiety” subgroup of MADRS. In fact, an anxiety node had positive connections with all MADRS items except *inability to feel*. In other words, anxiety have a profound effect on the symptoms of depression in some way. Anxiety may affect symptoms of depression by either worsening the symptom severity or changing the overall symptom network structure in psychopathological perspective, or by both. Please note the disease severity and global strength of network are not the same concepts (50). For instance, even though depressive symptoms are mild, the overall connectivity of the symptom network can be dense and strong (50). Analysis of general differences revealed the comorbid anxiety symptoms worsens the depression severity, and it is consistent with previous studies (51–53). However, NCT revealed there is no structural difference between networks of before and after PSM (*P* = .46 and *P = .46*). Although the global strength of the two networks showed statistically significant difference (*P = .02*) before PSM, this statistical significance did not persist after PSM suggesting that comorbid anxiety does not alter the overall strength of depression symptom network. This result suggests anxiety itself did not strengthen and increase the overall connectivity of depression symptoms. In conclusion, anxiety may affect depression in a way of worsening the severity of symptoms, rather than changing psychopathology.

Considering the low centrality of the vegetative group in the baseline network, the study suggests vegetative symptoms may be attributed largely to the anxiety component rather than depression, and therefore, treatment of anxiety may be the key to resolving such symptoms. Although there is no fixed guideline available for the depressive elderly with anxiety, selective serotonin reuptake inhibitors (SSRIs) are preferably used as first-line treatment due to their beneficial effects on somatic symptoms and low adverse effect rates (53, 54). Furthermore, their therapeutic influences on anxiety disorders as well as depression may augment the treatment effects on the anxious depression (55–57). Comparison of the centrality indices of the two networks after PSM decreased the gap between the two networks in all nodes, except for vegetative group. In other words, relative importance of vegetative symptoms increases with the presence of anxiety. Based on the previous study that mirtazapine [noradrenergic and specific serotonergic antidepressant (NaSSa)] appears to have similar effects on the anxious depression of the elderly to SSRIs (58) our finding suggests that mirtazapine may have treated the elderly with anxious depression by improving their vegetative symptoms (58–60).

This study has several limitations. First, the study involves restricted subject group. Symptom networks can be influenced by external factors such as age, gender, ethnicity and other socioeconomic factors. Therefore, the results may not be replicated in subjects manifesting different characteristics. For example, used dataset involved higher female subjects than males, and therefore, the results may specifically reflect the characteristics of females. However, elderly depression is particularly common in women (4), and therefore, our population reflect the reality of elderly depression. Also, the population of the used dataset includes Korean subjects exclusively. A study with a larger sample with more diverse characteristics is needed to determine the extent to which our results can be replicated. Second, this study was based on cross-sectional data, therefore, associations cannot be viewed as causal relationships. Whether correlations between symptoms are one-way or bi-directional causal relationships, or not causal at is unknown. Third, BAI score may be affected by associated somatic symptoms in late-life depression, so the efficacy of BAI in measuring the pure anxiety level of the depression is yet to be validated (61). However, network analysis may compensate the drawback of scale-based approach by finding informative or key symptoms rather than simply relying on total scores from scales (62, 63). Forth, self-reported method for assessing depression and anxiety in the elderly may lead to misinterpretation and make biased responses which may lower the precision of the analysis (61, 64). Fifth, the significant number of subjects were discarded during PSM, which is the main drawback of PSM methodology (65). Relatively small number of selected subjects (N = 199) might have increased statistical bias. Lastly, this study did not analyze the medication history of each patient, which may be a confounding factor.

## Conclusion

Based on the statistical analysis, anxiety worsens the severity of depression in the elderly. However, NCT after PSM revealed comorbid anxiety does not change the global structure and strength of the depression symptom network. Therefore, anxiety may affect LLD in a way of worsening the severity, rather than changing psychopathology. Additionally, the study revealed the centrality of vegetative symptoms was low in LLD but increased substantially in patients with comorbid anxiety.

## Data Availability Statement

The dataset for this study will not be made publicly available due to ethical restrictions. The dataset will be personally available if there is reasonable request.

## Ethics Statement

The studies involving human participants were reviewed and approved by Ajou University Hospital, institutional review of board. The patients/participants provided their written informed consent to participate in this study.

## Author Contributions

MA, SP, and HK conceived and designed the study. MA and SP performed the study and analyzed the data. MA, SP and HK wrote the manuscripts. SY and RP assisted on conducting network analysis. BP assisted with statistical analysis and data interpretation. SS and HK supervised on this project. HW analyzed the data and assisted in editing the manuscript. All authors contributed to the manuscript revision and approved to final draft of the manuscript before submission.

## Funding

This research was supported and funded by a grant from Health Fellowship Foundation, Republic of Korea and the Korean Health Industry Development Institute (grant No. HI19C0094).

## Conflict of Interest

The authors declare that the research was conducted in the absence of any commercial or financial relationships that could be construed as a potential conflict of interest.
