# Supplementary material for: Depressive Symptom Network Associated With Comorbid Anxiety in Late-Life Depression
Source: Front Psychiatry. 2019 Nov 20;10:856. doi: 10.3389/fpsyt.2019.00856 (PMC6880658; doi:10.3389/fpsyt.2019.00856)

**Supplementary Material**

**(Depressive Symptom Network Associated with Comorbid Anxiety in Late-life Depression)**

**Supplementary Table 1.** Mapping of Items in MADRS to DSM-5 Criteria

|  | **DSM-5 Criterion** |  | **MADRS Item** |
| --- | --- | --- | --- |
| Item | Description | Item | Description |
| A1 | Depressed mood or anhedonia | M1 | Apparent Sadness |
|  |  | M2 | Reported Sadness |
| A2 | Loss of interest or pleasure in most daily activities | M8 | Inability to Feel |
| A3 | Change in weight or appetite | M5 | Reduced Appetite |
| A4 | Insomnia or hypersomnia | M4 | Reduced Sleep |
| A5 | Psychomotor agitation or retardation | M3 | Inner Tension |
| A6 | Loss of energy or fatigue | M7 | Lassitude |
| A7 | Inappropriate guilt or sense of worthlessness | M9 | Pessimistic Thought |
| A8 | Impaired concentration or indecisiveness | M6 | Concentration Difficulties |
| A9 | Thoughts of death, suicidal ideation or suicidal attempt | M10 | Suicidal Thought |

MADRS indicates Montgomery–Åsberg Depression Rating Scale.

**Supplementary Figure 1.** Enrollment flow chart for of this study.


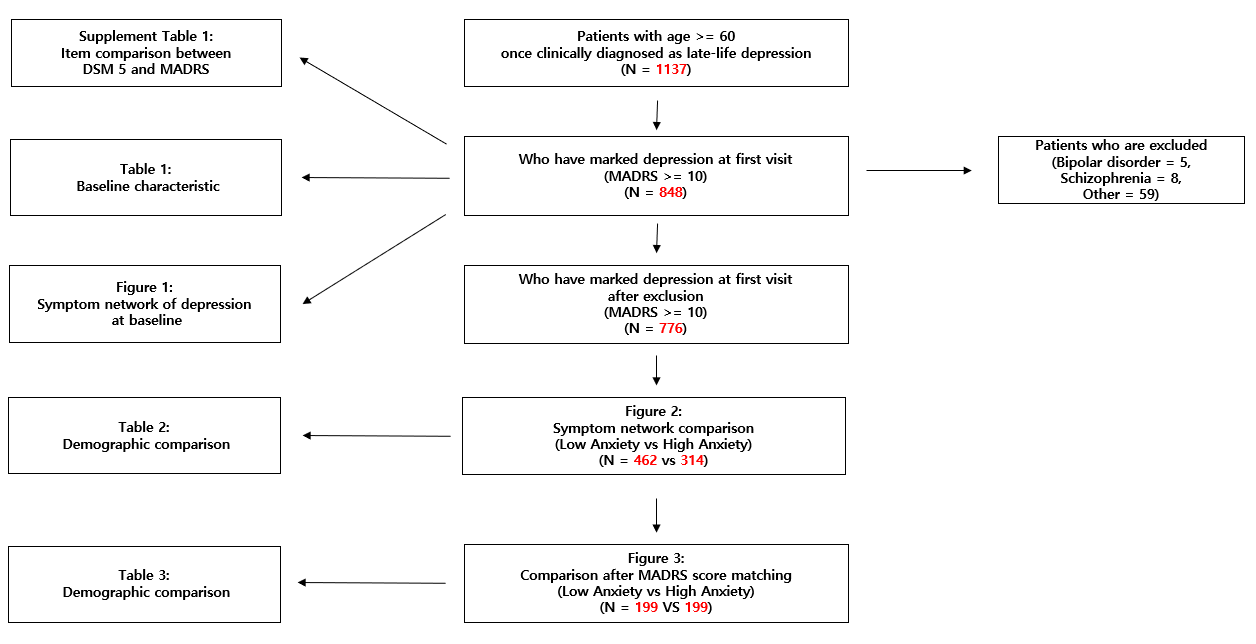


MADRS, Montgomery–Åsberg Depression Rating Scale

**Supplementary Figure 2.** Network Stability of Node Strength and Edge Weight Accuracy via Bootstrapping (Baseline network of late-life depression)


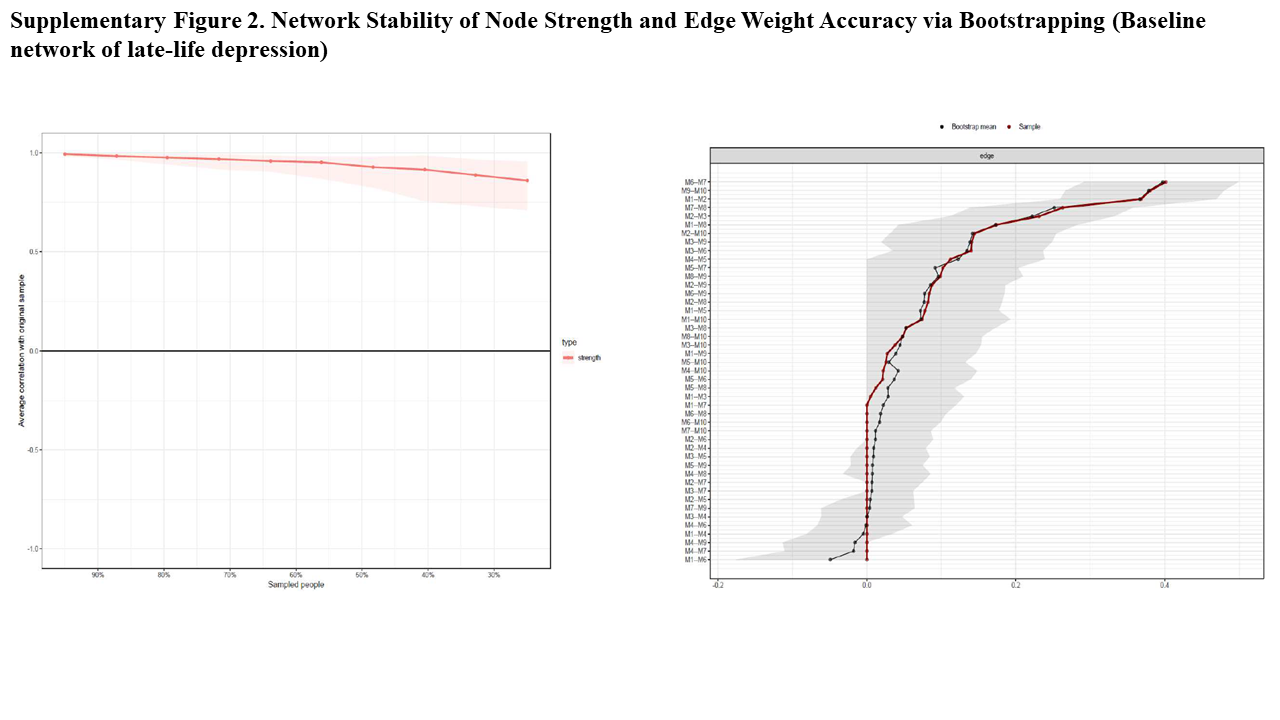


**Supplementary Figure 3.** Centrality indices of ‘anxiety node’-added depression symptom network.


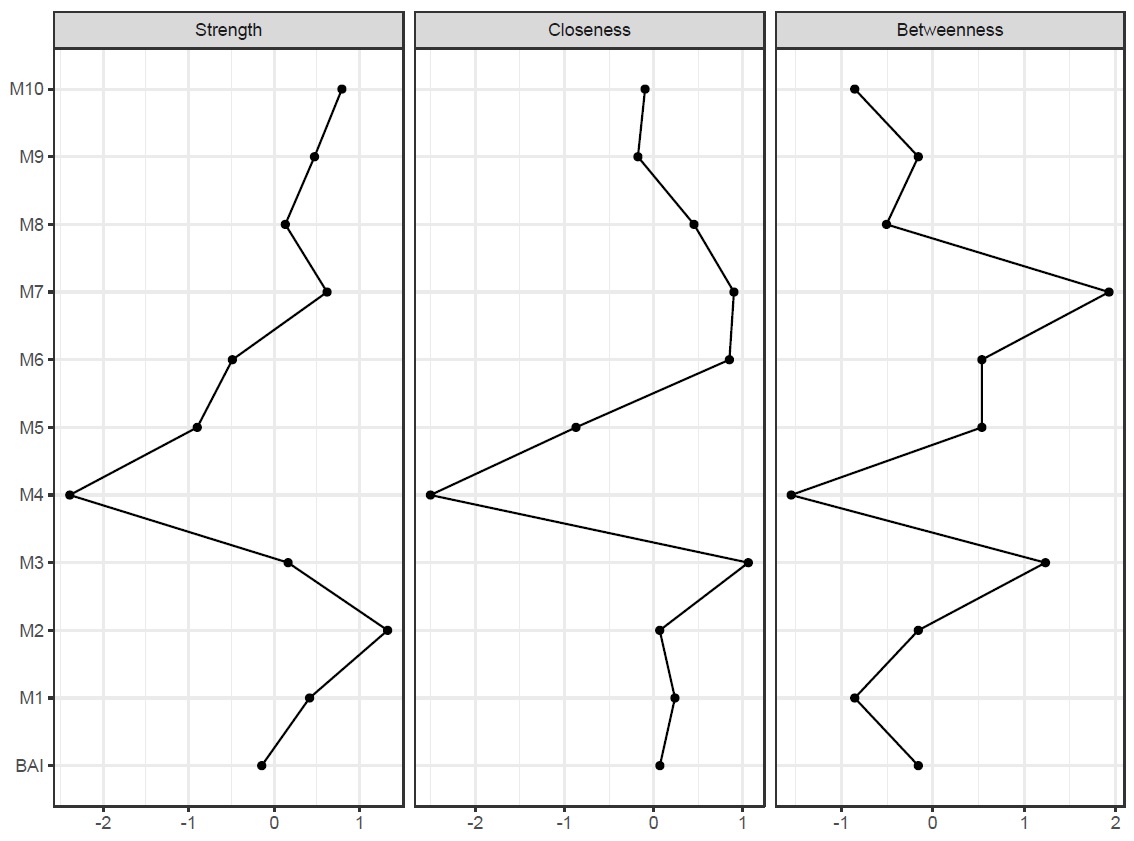


**Supplementary Figure 4.** Network Stability of Node Strength and Edge Weight Accuracy via Bootstrapping (‘Anxiety node’-added depression symptom network.)

**
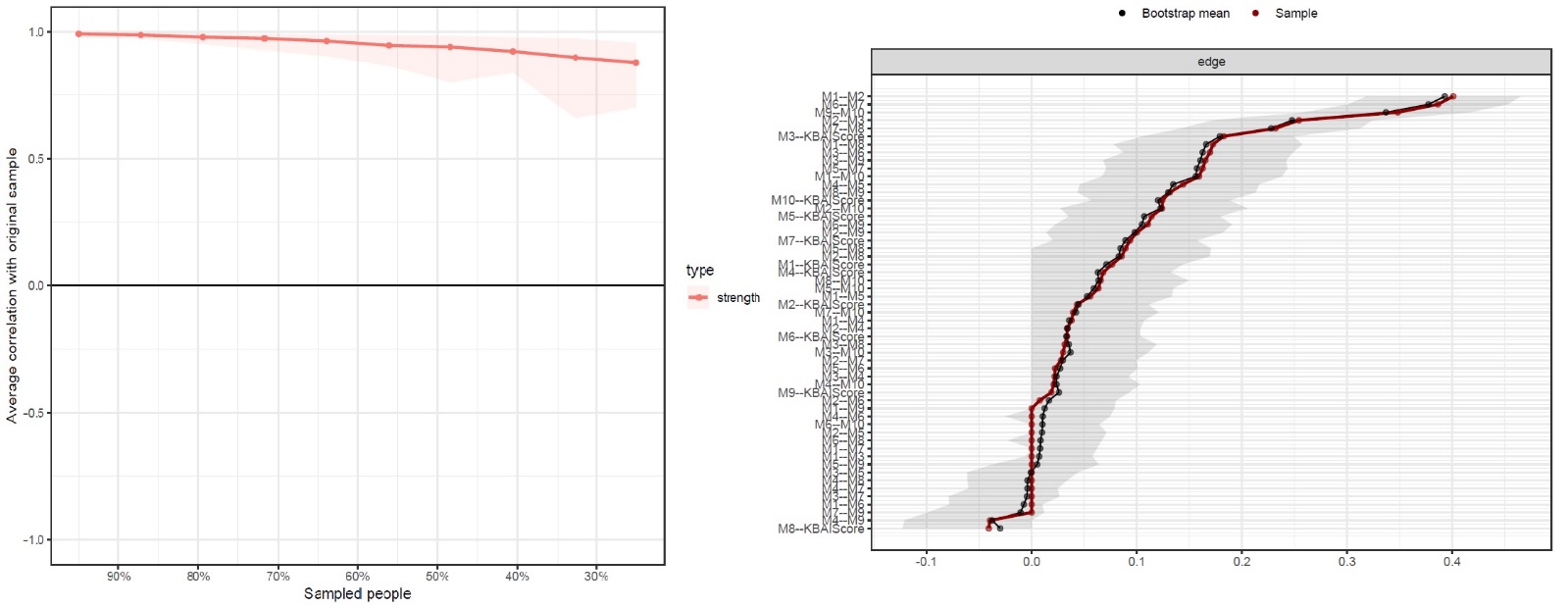
**

**Supplementary Figure 5.** Network Stability of Node Strength and Edge Weight Accuracy via Bootstrapping (The network of low vs high anxiety groups before and after propensity score matching)


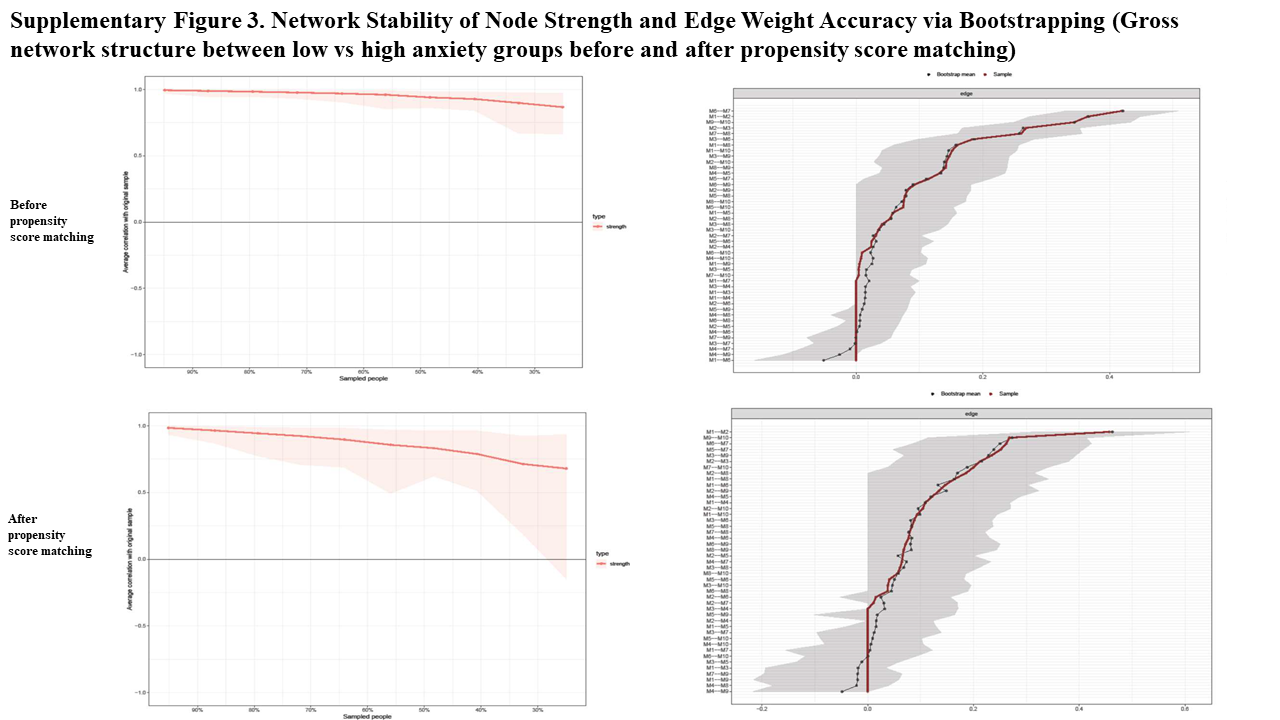

Supplement: Supplementary file 1 [file DataSheet_1.docx]
